# Prevalence and determinants of acute diarrhea among children younger than five years old in Jabithennan District, Northwest Ethiopia, 2014

**DOI:** 10.1186/s12889-017-4021-5

**Published:** 2017-01-19

**Authors:** Zelalem Alamrew Anteneh, Kassawmar Andargie, Molalign Tarekegn

**Affiliations:** 1 Department of Epidemiology, School of Public Health, College of Medicine and Health Science, Bahir Dar University, Bahir Dar, Ethiopia; 2 Department of Public Health, GAMBY College of Medicine and Health Sciences, Bahir Dar, Ethiopia; 3 Department of Research and Project Unit, Felege Hiwot Referral Hospital, Bahir Dar, Ethiopia

**Keywords:** Diarrheal Disease, Jabithennan District, Children younger than five years old

## Abstract

**Background:**

Despite the global decline in death rates of children younger than five years old, the risk of a child dying before turning five years of age remains highest in the WHO African Region. The problem of child death in Ethiopia is worse, with an Ethiopian child being 30 times more likely to die by his/her fifth birthday than a child in Western Europe. Therefore, the aim of this study was to assess the prevalence and factors associated with diarrhea among children younger than five years old.

**Methods:**

A community-based, cross-sectional study was conducted with mothers who had children younger than five years old from April to June 2014. A multistage sampling procedure was used to select eligible women. The data were coded, entered, cleaned and analyzed with the SPSS software package, version 16.

**Results:**

he data of 775 mothers were included in the analysis, and 21.5% of the children had diarrhea in the two weeks before the survey. The main factors affecting the occurrence of diarrhea were residence (Odds ratio (AOR) = 11.29, 95% Confidence interval (CI): 3.49-36.52), sex (AOR = 2.52, 95% CI:1.28-4.93), methods of complementary feeding (AOR = 50.88, 95% CI: 23.85- 108.54), types of water storage equipment (AOR = 19.50, 95% CI: 8.11-46.90), and cleansing materials used to wash hands (AOR = 5.53, 95% CI: 2.19-13.99).

**Conclusion:**

Approximately one-fifth of the children included in the study reported diarrheal disease. Residence, sex of the child, type of water storage container, methods of complementary feeding, and cleansing materials to wash the hands were the most important variables that affected the occurrence of diarrhea in children. Therefore, families, the government and nongovernmental organizations working in the area must cooperate in interventions and prevention to minimize the risk of disease.

**Electronic supplementary material:**

The online version of this article (doi:10.1186/s12889-017-4021-5) contains supplementary material, which is available to authorized users.

## Background

Despite the global decline in the death rates of children younger than 5 years old, the risk of a child dying before becoming 5 years of age remains highest in the WHO African Region (90 per 1000 live births), which is approximately seven times higher than that in the WHO European Region (12 per 1000 live births) [1].

Children in developing countries are disproportionately affected by preventable and treatable diseases with simple and affordable interventions. Consequently, children in these countries are 10 times more likely to die before the age of 5 years old than children in industrialized countries [2, 3].

People in the economically poorest regions of the world and the least developed countries continue to bear the heaviest burden of child deaths. More than four-fifths of all deaths among children younger than 5 years old in 2011 occurred in sub-Saharan Africa and South Asia [4]. The problem in Ethiopia is even worse than elsewhere in the world, with an Ethiopian child being 30 times more likely to die by his/her fifth birthday than a child in Western Europe [3].

Diarrheal diseases account for 1 in 9 child deaths worldwide, making diarrhea the second leading cause of death among children younger than the age of five [5]. The disease is preventable and is characterized by the passage of loose or watery stools three or more times over a 24-h period [6].

Diarrhea is responsible for 17% of all deaths (approximately 2.5 million deaths each year) among children younger than 5 years old worldwide; this rate is higher than that of AIDS, malaria, and measles combined. The majority (42%) of these deaths are concentrated in the Sub-Saharan African countries, including Ethiopia (88 per 1000 live births) [7].

Recent national estimates have indicated that the two-week prevalence of diarrhea in children was approximately 13% [8]. Moreover, few local studies have reported that the magnitude of diarrhea among children younger than 5 years old in different regions of the country ranges from 18 to 31% [9–12]. Although this evidence is available, there has still been a lack of studies in the country to obtain up-to-date information on the disease and to prioritize interventions by decision makers to overcome the problem. Therefore, the aim of this study was to identify the magnitude and to determine the factors that affect diarrheal disease among children younger than five years old.

## Methods

A community-based, cross-sectional study was conducted among mothers with children younger than 5 years old from April to June 2014in Jabitehnan District, West Gojjam Zone, Amhara Region, Ethiopia. This District is located 390 kilometers northwest of Addis Ababa; it has 37 rural and 2 urban kebeles.

According to a 2014 Amhara Bureau of Finance and Economic Development office report, the total population of the District was estimated to be 207,162. The number of childbearing women (15-49 years old) was 45,938, and the estimated number of children younger than 5 years old in the district was 27,759.

### The eligibility criteria

Mothers who had children younger than 5 years old living in the selected kebeles of the District in the last 6 months of the survey.

### Sample size determination of the study

The sample size of the study was determined using a single population proportion formula, considering an estimate of 18% expected prevalence of diarrhea among children younger than 5 years old [13]. Assuming any particular outcome to be within a 4% marginal error and a 95% confidence interval of certainty, the final sample size with a design effect of 2 and a 10% non response rate was determined to be 781 mothers of children younger than 5 years old.

### Sampling procedure

**Fig. 1:**
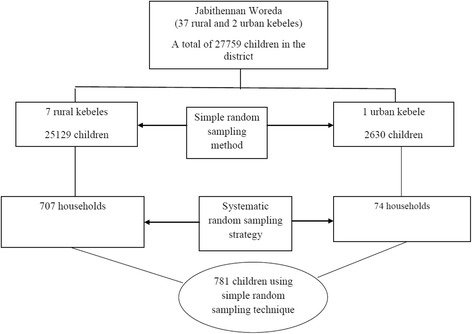
Schematic presentation of the sampling frame for Children Younger than five years old in Jabithennan District, West Gojjam Zone, June 2014

A multistage sampling technique was used to select the study participants. Initially, the study area was divided into two strata: urban and rural kebeles. One urban and seven rural kebeles were selected by a simple random sampling technique. The calculated sample size was allocated into randomly selected kebeles in the district, proportional to the size of the population. The allocated sample size in each kebele was divided by the number of households in the kebele with children younger than five years old to obtain a sampling fraction. Then, a systematic sampling technique was employed to obtain the respondents to the study. In cases in which more than one mother with children younger than 5 years old was found in the household, a simple random sampling method was employed to select the mother to be included, and in cases of more than one children younger than 5 years old of one mother in the household, the eldest child was studied (Fig. 1 shows the schematic presentation of smapleling procedure).

### Variables

#### Dependent variable

Diarrheal disease.

### Independent variables

#### Socio-demographic variables

Age of mother, educational status of mother, family size, residential area, sex of child, age of child, number of children younger than 5 years old in the family, etc.

### Environmental variables

Latrine availability, hand washing facility, source of water, etc.

### Behavioral variables

Latrine utilization, hand washing practices, exclusive breastfeeding, complementary feeding practices of children, etc.

### Data collection and analysis

The data were collected using a face-to-face administrated questionnaire and an observation checklist; the questionnaire was adapted from reviewed articles [9, 11, 14] (Additional file 1). The data were collected by six diploma nurses who had experience in data collection in the rural community

Two days of training were provided to maintain the quality of the data and ethical issues. A pre-test was performed in kebeles not included in the main survey, and the necessary corrections were performed to the tool before the data collection was undertaken. The collected data were checked for errors and incompleteness on a daily basis. Finally, the data were coded, entered and analyzed using SPSS software, version 16 (Chicago, IL, USA).

Descriptive statistics were used to determine the magnitudes of events in the study. Bivariate logistic regression analysis was computed to test whether there were associations between diarrheal disease and selected predictor variables.

Factors associated with an outcome variable in bivariate analysis at a significance level of 0.20 were identified and exported to multivariable logistic regression analysis, and the model was built with backward elimination. Regarding the effect of multi collinearity, intra-predictor variables were tested using the standard errors of the beta coefficients. The assumptions of logistic regression analysis were assessed using the Hosmer-Lemeshow model fitness test.

Finally, 95% confidence intervals not including one, with their corresponding P-values less than 0.05, were considered statistically significant.

### Operational definitions

#### Diarrheal Disease (the outcome of the study)

The occurrence of loose or watery diarrhea at least three times per day for the previous 2 weeks.

### Hand washing at critical times

Washing the hands before and after cooking foods, after latrine use, and before feeding the child.

### Proper hand washing

Hand washing with soap or ash at critical times.

### Kebele

The lowest governmental administrative unit in the hierarchy.

## Results

### Socio-demographic characteristics of the study participants

The data of 775 mothers of children younger than five years old were included in the analysis of the intended sample size of 781 mothers, resulting a response rate of 99.2%. Five hundred sixty- nine (73.4%) mothers were in the age range of 20-34 years old, and the mean age of the respondents was 30.04 years old, with a standard deviation of 5.19 years.

Four hundred two (51.9%) of the children younger than 5 years old were female, and regarding religious affiliation and the residences of the respondents, 766 (98.8%) were Orthodox Christians, and 708 (91.4%) were from rural kebeles.

Concerning the number of children younger than 5 years old in the sampled households, 637 (82.2%) of them reported having one child, whereas the remainder, 138 (17.8%) households, had two children.

Seven hundred fifty-two (97%) of the respondents were married, and 501 (64.6%) of the households had a family size less than five members. Regarding the occupation and educational status of the husbands of the study participants, 737 (95.1%) engaged in farming, and 87 (11.7%) attended primary school (see Table 1).

**Table 1:** Socio-demographic characteristics of mothers with children younger than five years old in West Gojjam Zone, Jabithenna District, June 2014

Variables	Response category	Frequency	Percentage
Age	<20	34	4.4
	20-34	569	73.4
	35-59	172	22.2
Residence	Rural	708	91.4
	Urban	67	8.6
Religion	Orthodox	766	98.8
	Muslim	9	1.2
Number of family members	<5	501	64.6
	> = 5	274	35.4
Number of children younger than five years old	One	637	82.2
	Two	138	17.8
Age of the child (months)	<6	117	15.1
	6-11	129	16.6
	12-23	198	25.5
	24-35	186	24
	36-47	110	14.2
	48-59	35	4.5
Sex of child	Male	402	51.9
	Female	373	48.1
Educational status of mother	Not able to read and write	532	68.6
	Can read and write	155	20.0
	Primary	62	8.0
	Secondary	17	2.2
	College or above	9	1.2
Maternal occupation	Farmer	737	95.1
	Daily laborer	5	0.6
	Merchant	1	0.1
	Governmental employee	18	2.3
	Housewife	14	1.8
Marital status	Married	752	97
	Single	1	0.1
	Divorce	19	2.5
	Widowed	3	0.4
Occupation of husband	Farmer	680	90.3
	Daily laborer	33	4.4
	Governmental employee	16	2.1
	Merchant	24	3.2
Education of the husband	Not able to read and write	371	49.3
	Read and write	257	34.1
	Primary	87	11.6
	Secondary	24	3.2
	College or above	14	1.9
Household average monthly income	<500	148	19.1
	500-1000	320	41.3
	>1000	307	39.6

### Environmental characteristics of the respondents

According to the findings of this study, approximately one-fifth of the households were drinking water from unprotected sources. Regarding the required time spent to obtain drinking water,192 (24.8%) and 173 (22.3%) of households had spent 15 to 30 and more than 30 min walking distances to fetch water, respectively. Four hundred twenty-eight (55.2%) of the households used a jerry can (narrow-neck water container) to store drinking water.

Seven hundred thirty-four (94.7%) of the households had a latrine, and 725 (98.8%) of the latrines were traditional pit latrines.

More than 30% of the latrines in the district did not have a hand washing facility. According to this study, 216 (29.4%) of the latrines had feces around their holes. In addition, there were observable feces in the compounds of 98 (12.6%) households included in the study. This study also showed that 194 (25%) of the households shared the same house with domestic animals. Regarding the waste disposal methods of the households, 308 (39.7%) and 207 (34.8%) did not have proper solid and liquid waste disposal systems, respectively (see Table 2).

**Table 2:** Environmental characteristics of households in West Gojjam Zone, Jabithennan District, June 2014

Variables	Response category	Frequency	Percentage
Source of water	Protected source	624	80.5
	Unprotected water source	151	19.5
Time taken to fetch water	<15 min	410	52.9
	15-30 min	192	24.8
	>30 min	173	22.3
Type of water storage	Jerrican (narrow neck)	428	55.2
	Not narrow	347	44.8
Separate container of drinking water	Yes	696	89.8
	No	79	10.2
Availability of latrine	Yes	734	94.7
	No	41	5.3
Type of latrine	Traditional	725	98.8
	VIP latrine	1	0.1
	Communal	8	1.1
Years latrine constructed	≤2 years	159	21.7
	>2 years	575	78.3
Function of latrine	Functional	708	96.5
	Non-functional	26	3.5
Availability of hand washing	Yes	425	68
	No	309	42
Location of hand washing facilities	Next to latrine	355	83.5
	Within walking distance	70	16.5
Frequency of latrine utilization	Rarely	55	7.4
	Mostly	43	5.8
	Always	625	83.1
	Summer season only	11	1.7
Feces around in the latrine hole	Yes	216	29.4
	No	518	70.6
Presence of feces in the compound	Yes	98	12.6
	No	677	87.4
Animals in the house	Yes	194	25
	No	581	75
Type of solid waste disposal	Open field	308	39.7
	Safe disposal	467	60.3
Type of liquid waste disposal	Open field	270	34.8
	Constructed pit	505	65.2

### Prevalence of diarrheal disease and behavioral and child care practices

Regarding the time when breastfeeding started after childbirth, 190 (24.5%) of the children had received breast milk after one hour, and approximately one in five children of the respondent mothers were not fed colostrum.

Concerning the initiation time of complementary feeding, 188 (29.9%) of the mothers started feeding additional food to their children after six months of age. The study further indicated that 199 (25.7%) and 447 (74.3%) of the mothers fed their children by bottle and cup feeding, respectively, as a complement to breast milk.

Regarding the hand washing practices of mothers, 237 (27.4%) of them did not wash their hands at the five critical times. Of the total mothers who reported washing hands at the five critical times 495 (63.9%) of them washed their hands without any detergent. In addition, 595 (96.9%) and 624 (92.4%) of the children were vaccinated for measles and received vitamin A supplementation, respectively. The findings of this study declared that the prevalence of diarrheal disease among children younger than 5 years old was 167 (21.5%) (see Table 3).

**Table 3:** Behavioral and child care practices of mothers in Jabithennan District, West Gojjam Zone, June 2014

Variables	Response category	Frequency	Percentage
Time of breast milk feeding initiation	Within one hour	585	75.5
	After one hour	190	24.5
Feeding of infant with colostrum of breast milk	Yes	625	80.6
	No	150	19.4
Age at complementary/additional food started	At 6 months	455	70.4
	Less than 6 months	3	0.4
	More than 6 months	188	29.2
Equipment of complementary feeding (646)	Bottle	199	25.7
	Cup	447	74.3
Hand washing practices/habits	At all five critical times	563	72.6
	Not all five critical times	237	27.4
Cleansing materials used to wash hands	Soap with water	268	34.6
	Ash with water	12	1.5
	Water only	495	63.9
Measles vaccination (614)	Yes	595	96.9
	No	19	3.1
Vitamin A (675)	Yes	624	92.4
	No	51	7.6
Had diarrhea	Yes	167	21.6
	No	608	78.4

### Predictors of diarrheal disease among children younger than 5 years old in Jabithennan District, West Gojjam Zone, June 2014

In the bivariate logistic regression analysis between different predictor variables and diarrheal disease in the previous 2 weeks, the age of the mother, residence, number of children younger than 5 years old in a family, sex of the child, time taken to fetch water, type of water storage, availability of a latrine, availability of feces in the compound, domestic animals sharing the same house, time that breastfeeding started, colostrum breastfeeding, methods of complementary feeding and cleansing materials used to wash hands were found to show associations at P-values less than or equal to 0.2.

However, only residence, sex of the child, time taken to fetch water, type of water storage, methods of complementary feeding and cleansing materials used to wash hands appeared in the final condensed model of the multivariable analysis (see Table 4).

**Table 4:** Multivariate logistic regression analysis of diarrheal disease with selected predictor variables among children younger than five years old in Jabithennan District, West Gojjam Zone, in June 2014

Variables	Presence of diarrhea		COR (95% CI)	AOR (95%CI)
	Yes	No		
Residence (*n* = 775)				
Rural	159	549	2.136 (1-4.56)	11.29 (3.49-36.52)
Urban	8	59	1	1
Sex (*n* = 775)				
Male	101	301	1.56 (1.10-2.21)	2,52 (1.28-4.93)
Female	66	307	1	1
Time taken to fetch water				
<15 min	80	330	1	1
15-30 min	40	152	1.08 (0.70-1.66)	0.38 (0.16-0.88)
>30 min	47	126	1.53 (1.01-2.32)	0.73 (0.31-1.71)
Types of water storage (*n* = 775)				
Jerrican (narrow neck)	9	419	1	1
Not narrow	158	189	38.91 (19.45-77.84)	19.50 (8.11-46.90)
Methods of complementary feeding (*n* = 646)				
Bottle	137	62	59.52 (33.25-106.54)	50.88 (23.85-108.54
Cup	16	431	1	1
Cleansing materials used to wash hands (*n* = 775)				
Soap and water	13	255	1	1
Ash and water	3	9	6.53 (1.58-27.06)	0.84 (0.09-7.55)
Water only	151	344	8.61 (4.77-15.52)	5.53 (2.19-13.99)

## Discussion

The prime objectives of this study were to identify the prevalence and determinants of diarrheal disease among children younger than 5 years old. It was found that more than one-fifth (21.5%) of the children included in the study reported diarrheal disease in the 2 weeks before the survey. This finding was supported by a study performed in Kashmir, India, where the prevalence of diarrhea among children younger than 5 years old was 25.2% [15]. The current finding was also in agreement with studies conducted in Nakemet, Western Ethiopia, and Jigjiga District, Somali Region, Eastern Ethiopia, where the rates of diarrhea among children younger than 5 years old in these two regions were 28.9% and 27.3% respectively [13, 16].

However, the current finding was higher than the finding of the Ethiopian demographic and health survey 2011 (EDHS),in which the magnitude of diarrheal disease among children younger than 5 years old was 13% [17], and the current finding also higher than a finding from Mecha district, West Gojjiam, Ethiopia, where the prevalence of diarrhea was 18% [18].

However, the current findings were lower than those of similar studies conducted in rural households in Northwestern Burundi, where the prevalence of diarrhea was 32.6% [19], and of a study conducted in Arba Minch district, south Ethiopia, where the prevalence of diarrhea reported was 30.5% [20]. The difference could be attributed to the sample size, study period, environmental conditions and socio-economic and cultural differences.

Children from rural communities were more than eleven times more likely to report diarrheal disease, compared to children from urban areas. This finding was supported by a similar study conducted in Sheko district, Southwest Ethiopia, and DebreBirehan Town, North East Ethiopia, where children from families in urban residences were affected by diarrhea [21, 22]. This finding could be attributed to different factors, including access to a safe and adequate water supply, the literacy status of the people, knowledge of hygienic activities and communicable disease prevention and control practices, and to latrine availability being severely limited in rural areas of the country.

The gender of the child was a statistically significant predictor of childhood diarrhea, with boys more than two times more likely to be affected by diarrheal disease than girls. This finding was supported by other studies conducted in Sudan and Nigeria [23, 24].

Although the reason why boys younger than 5 years old were more likely to be affected by diarrheal disease than their female counterparts is beyond the scope of this study, the probable reason is that, in Ethiopia, playing outside of home is allowed for boys, and they begin to participate in economic activities, such as tending to domestic animals in the field when they reach 4 to 5 years of age with their elders; in contrast, girls are not allowed to be involved in out-of-home activities, and they are not permitted even to play outside of one’s own compound [25]. This difference might have contributed to boys having a greater opportunity to wander off into unsanitary surroundings than girls, eventually leading to diarrheal morbidity. This finding was supported by a study conducted in pediatric hospital admissions in Hong Kong: Hon and Nelson (155) reported that boys had a consistent excess in admissions, compared to girls [23].

In addition, children from households using broad-necked equipment for water storage were at more than 19 times greater risk of diarrhea, compared to children from households using jerry cans (narrow-necked water container). This finding was supported by different studies performed in Lalit pur district, Nepal, Kersa district, Eastern Ethiopia, and Debrebirehan town, Northeast Ethiopia [26–28]. This finding was absolutely the result of water being contaminated at or following collection during transportation and/or storage since the water storage containers are uncovered and broad necked, so they can be contaminated easily by pets, dirt, or other debris [13].

The findings of this study were also supported by a Centers for Disease Control and Prevention report that found that microbial contamination of water was usually associated with storage vessels having wide openings, such as buckets and pots, which are vulnerable to the introduction of hands, as well as cups and dippers, which can easily carry contamination to the water [29].

In this particular study, it was found that the odds of diarrheal disease were much higher among children who received bottle methods of complementary feeding, compared to cup methods of feeding. This finding was in accordance with other studies in Nekemit town, Western Ethiopia [13], and Gondar, Northwest Ethiopia [30],where the bottle method of complementary feeding was more likely to be associated with diarrheal diseases. This finding might be associated with improper cleansing of bottles.

Moreover, children of mothers who washed their hands using water only at critical times were more than five times more likely to report diarrhea, compared to children of mothers who washed their hand using water and soap.

The finding was supported by similar studies conducted in Bangladesh [17, 31] and was also supported by similar studies performed Jigjiga District, Somali Region, Eastern Ethiopia, and in Sheko district, Southwest Ethiopia, where mothers’ hand washing habits affected the occurrence of diarrheal disease among their children [16, 21].

## Conclusion

The findings of this study revealed that one out of five children had diarrhea in the Jabithennan district, and several factors were found to affect diarrheal disease among children in the district. Therefore, the government, families and nongovernmental organizations working with children and mothers must cooperate regarding interventions to minimize the risks of the disease.

The regional health bureaus and nongovernmental organizations should address the availability of safe drinking water and the sanitation of rural communities, which might be contributing to the occurrence of diarrheal disease among children.

The local government should focus on the accessibility of safe drinking water for the community. In addition, regional and local health officials must work to create awareness about the type of water storage containers that the community should use. Moreover, all mothers should be informed that the cup method of complementary feeding is better than bottle feeding to prevent diarrhea, and concerned bodies must work to create awareness about how mothers use and access hand washing detergents during critical times.

## Additional file

Additional file 1: The English version questionnaires. (DOCX 26 kb)

## References

[1] World health organization. Global Health Observatory (GHO). Under-five mortality. Available from: http://www.who.int/gho/en/. Accessed 12 Jan 2017.

[2] WHO: Child mortality. Updated September 2011. Available from: http://www.who.int/pmnch/media/press_materials/fs/fs_mdg4_childmortality/en/. Accessed 10 Nov 2014.

[3] UNICEF: Reduce child mortality; Millennium development Goals. Available from: http://www.unicef.org/mdg/childmortality.html. Accessed 10 Nov 2014.

[4] Children: reducing mortality [Internet]. World Health Organization. 2017 [cited 12 January 2017]. Available from: http://www.who.int/mediacentre/factsheets/fs178/en/.

[5] CDC: Global water, Sanitation and hygiene (WASH). Global diarrheal burden. Available from: http://www.cdc.gov/healthywater/global/diarrhea-burden.html. Accessed 10 Nov 2014.

[6] Gerald T. Keusch, Olivier Fontaine, Alok Bhargava, Cynthia Boschi-Pinto, Zulfiqar A. Bhutta, Eduardo Gotuzzo et al. Disease Control Priorities in Developing Countries. 2nd edition. Washington (DC): The International Bank for Reconstruction and Development / The World Bank; 2006. Chapter 19. Available from: https://www.ncbi.nlm.nih.gov/books/NBK11764/. Co-published by Oxford University Press, New York. Accessed 12 Nov 2014.

[7] Liu L, Johnson HL, Cousens S, Perin J, Scott S, Lawn JE (2012). Child Health Epidemiology Reference Group of WHO and UNICEF. Global, regional, and national causes of child mortality: an updated systematic analysis for 2010 with time trends since 2000. Lancet.

[8] Central Statistical Agency [Ethiopia] and ICF International (2011): Ethiopia Demographic and Health Survey. Addis Ababa, Ethiopia and Calverton, Maryland, USA: Central Statistical Agency and ICF International; 2012.

[9] Muluken D, Abera K, Worku T (2011). Predictors of under-five childhood diarrhea : Mecha District, West Gojam Ethiopia. Ethiop J Health Dev.

[10] Shikur M, Marelign T, Dessalegn T (2013). Morbidity and associated factors of diarrhea diseases among under- five children in Arba-Minch district, Southern Ethiopia. Sci J Public Health.

[11] Bezatu M, Yemane B, Alemayehu W (2013). Prevalence of diarrhea and associated risk factors among children under-five years of age in Eastern Ethiopia: A cross-sectional study. J Prev Med.

[12] Amare D, Fasil T, Belaineh G (2007). Determinants of under-five mortality in Gilgel Gibe Field Research Center, Southwest Ethiopia. Ethiop J Health Dev.

[13] Birke WA (2008). stepwise regression analysis on under-five diarrheal morbidity prevalence in Nekemte town, western Ethiopia. East Afr J Public Health.

[14] Gebru T, Taha M, Kassahun W. Risk factors of diarrhoeal disease in under-five children among health extension model and non-model families in Sheko district rural community, Southwest Ethiopia: comparative cross-sectional study. BMC Public Health. 2014;14:395. doi:10.1186/1471-2458-14-395. 10.1186/1471-2458-14-395 PMC4031974 24758243

[15] Ahmed S, Farheen A, Muzaffar A (2008). Prevalence of diarrhoeal disease, its seasonal and age variation in under- fives in Kashmir India. Int J Health Sci.

[16] Abdiwahab Hashi, Abera Kumie, Janvier Gasana. Prevalence of Diarrhoea and Associated Factors among Under-Five Children in Jigjiga District, Somali Region, Eastern Ethiopia. Open J Prev Med. 2016;6(10):233–46. doi:10.4236/ojpm.2016.610022.

[17] Baker KK, Fahmida Dil F, Farzana F, Shahnawaz A, Sumon Kumar D, Faruque ASG (2014). Association between moderate-to-severe diarrhea in young children in the global enteric multicenter study (GEMS) and types of hand washing materials used by caretakers in Mirzapur, Bangladesh. Am J Trop Med Hyg.

[18] Dessalegn M, Kumie A, Worku W (2011). Predictors of under-five childhood diarrhea: Mecha District, West Gojam, Ethiopia. Ethiop. J. Health Dev..

[19] Katharina D, Patrik T, Jochen R, and Michael M. Diarrhoea prevalence in children under five years of age in rural Burundi: an assessment of social and behavioural factors at the household level. Glob Health Action. 2014; 7: 24895. http://dx.doi.org/10.3402/gha.v7.24895. 10.3402/gha.v7.24895 PMC4141944 25150028

[20] Shikur Mohammed and Dessalegn Tamiru. The burden of diarrheal diseases among children under five years of age in Arba Minch District, Southern Ethiopia, and Associated Risk Factors: A Cross-Sectional Study. International Scholarly Research Notices Volume 2014, Article ID 654901, 6 pageshttp://dx.doi.org/10.1155/2014/654901. 10.1155/2014/654901 PMC4897213 27433486

[21] Gbru T, Tasha M, Kassahun W. Risk factors of diarrheal disease in under-five children among health extension model and non-model families in Sheko district rural community, Southwest Ethiopia: comparative cross-sectional study. BMC Public Health. 2014;23(14):395. doi:10.1186/1471-2458-14-395. 10.1186/1471-2458-14-395 PMC4031974 24758243

[22] Mamo A,Hailu A . Assessment of Prevalence and Related Factors of Diarrheal Diseases among Under-Five Year’s Children in Debrebirehan Referral Hospital, Debrebirehan Town, North Shoa Zone, Amhara Region, Ethiopia. Open Access Library Journal 2014;1 e283. Doi: http://dx.doi.org/10.4236/oalib.1100283.

[23] Siziya S, Muula AS, Rudatsikira E (2013). Correlates of diarrhea among children below the age of 5 years in Sudan. Afr Health Sci.

[24] Yilgwan CS, Okolo SN (2012). Prevalence of diarrhea disease and risk factors in Jos University Teaching Hospital, Nigeria. Ann Afr Med.

[25] UNICEF: Child Protection. Available from:www.unicef.org/ethiopia/Chapter_4_(72dpi).pdf). Accessed 12 Nov 2014.

[26] Karki T (2010). Factors related to the occurrence of diarrheal disease among under-five children in Lalitpur district of Nepal. J Pub. Health Dev.

[27] Mengistie B, Berhane Y, Worku A (2013). Prevalence of diarrhea and associated risk factors among children under-five years of age in Kersa district Eastern Ethiopia. Open J Prev Med.

[28] Mamo A, Hailu A (2014). Assessment of prevalence and related factors of diarrheal diseases among under-five year’s children in Debrebirehan Referral Hospital, Debrebirehan Town, North Shoa Zone, Amhara Region, Ethiopia. OALib J.

[29] Safe Water Storage | The Safe Water System | CDC [Internet]. Cdc.gov. 2017 [cited 12 January 2017]. Available from: https://www.cdc.gov/safewater/storage.html.

[30] Lengerh A, Moges F, Unakal C, Anagaw B (2013). Prevalence, associated risk factors and antimicrobial susceptibility pattern of Campylobacter species among under five diarrheic children at Gondar University Hospital, Northwest Ethiopia. BMC Pediatr.

[31] Luby SP, Halder AK, Huda T, Unicomb L, Johnston RB (2011). The effect of hand washing at recommended times with water alone and with soap on child diarrhea in Rural Bangladesh: an observational study. PLoS Med.

