# Supplementary material for: Prevalence and determinants of acute diarrhea among children younger than five years old in Jabithennan District, Northwest Ethiopia, 2014
Source: BMC Public Health. 2017 Jan 19;17:99. doi: 10.1186/s12889-017-4021-5 (PMC5248477; doi:10.1186/s12889-017-4021-5)
Supplement: Additional file 1: — The English version questionnaires. (DOCX 26 kb) [file 12889_2017_4021_MOESM1_ESM.docx]

**The English Version questionnaires**

**Part I: Socio-demographic information**

| *Codes* | *Questions* | *Responses* | *Skip* |
| --- | --- | --- | --- |
| 101 | What is your age in years?  (Age of the respondent ) | --------------- |  |
| 102 | Where is your place of residence? | 1.Rural  2.Urban |  |
| 103 | What is your Religion? | 1. Orthodox  2. Muslim  3. Protestant  99. Others/ specify ------ |  |
| 104 | How many family members do you have? | ---------------- |  |
| 105 | How many under five children do you have? | 1. One  2. Two and above | 108 |
| 106 | Age of child (elder one)  If more than one child | ----------------months |  |
| 107 | Sex of child elder one  If more than one child | 1.Male  2.Female |  |
| 108 | Dose the child had diarrhea in the last two weeks? | 1. 1. Yes 2. 2. .No |  |
| 109 | What is your Education level? | 1.Unable to read and write  2.read and write  3.primary education  4.secondary education  5.college and above |  |
| 110 | What is your occupation? | 1. Farmer  2. Daily laborer  3. Merchant  4.Governmental employee  99. Others / specify ----------- |  |
| 111 | What is your marital status? | 1. Married  2.Unmarride  3. Divorced  4. Widowed | 114 |
| 112 | What is your spouse’s occupation? | 1. Farmer  2. Daily laborer  3. Merchant  4. Government Employee  99.Other/specify----------- |  |
| 113 | What is your spouse’s education level? | 1 .Unable to read and write  2.read and write  3.primary education  4.secondary education  5.college and above |  |
| 114 | What is your average monthly income per month (in Ethiopian birr) | -------------------- |  |

**Part II: Environmental and behavioral related factor**

| Codes | questions | Responses | Skip |
| --- | --- | --- | --- |
| 201 | What is the main source of water for the household? (More one answer is possible) | 1. Protected well /spring  2. Unprotected well/spring  3. River water  4. Rain water  5. pipe line  99. Others /specifies---------- |  |
| 202 | How much minutes are needed to collect water (to go back and forth)? | 1. 1. Less than 15 minutes 2. 2. 15-30 Minutes 3. 3. More than 30 minutes |  |
| 203 | What container do you use for water storage at home? | 1. 1.Pot c/clay pot 2. 2.Bucket 3. 3. Jerrican 4. 4. Guard 5. 99..others/specify----------- |  |
| 204 | Did you use separate container for storage of drinking water? | 1.Yes  2.No |  |
| 205 | If the water storage container is pot and bucket what about the presence of scooper or dipper? | 1. 1.Present 2. 2. Absent |  |
| 206 | If the water storage container is pot and buckets what about scooper or dipper? | 1. Any  2. Separate |  |
| 207 | Do you have latrine? | 1.Yes  2. No | 218 |
| 208 | What type of latrine do you have? | 1. Traditional Pit latrine  2. VIP latrine  3. Communal latrine  99. Others/specify |  |
| 209 | How many years since latrine is  Constructed? | Specify in year /month/____/____ |  |
| 210 | Is the latrine functional? | 1.Yes  2. No |  |
| 211 | Observe | 1.Functional  2.Not functional |  |
| 212 | Is the latrine has hand washing facilities? | 1.Yes  2.No | 218 |
| 213 | Observe | 1.Available  2. Not available |  |
| 214 | How close are hand-washing facilities to the latrine? | 1. 1.Next to the latrine 2. 2.Within walking distance |  |
| 215 | How frequently is the latrine used? | 1.Rarely  2.Mostly  3.Always  4.summer |  |
| 216 | Are there feces around the hole of the latrine? | 1.Yes  2.No |  |
| 217 | Observe | 1.Available  2. Not available |  |
| 218 | Are there any feces in the compound? | 1.Yes  2. No |  |
| 219 | Observe | 1.Available  2. Not available |  |
| 220 | Are you keeping domestic animals with you in the same house? | 1. Yes  2. No |  |
| 221 | Where do you dispose solid wastes? (more than one possible answer) | 1. Open field  2. Burning  3. Properly constructed dug  4. Compost  99. Others /specify---- |  |
| 222 | Where do you dispose liquid wastes? | 1. In the field  2. Properly constructed site  99. Other /specify ----- |  |

Part III: Behavioral Factors related questions

| Codes | Questions | Responses | Skip |
| --- | --- | --- | --- |
| 301 | When did you start to feed your breast for your infant after delivery? | 1. within 1 hour  2. After 1 hour |  |
| 302 | Did you feed infants with colostrums of breast milk? | 1. yes  2. No |  |
| 303 | Did you start complementary food including water? | 1. 1.yes 2. 2. No | 306 |
| 304 | When should children start complementary feeding? | -----------months |  |
| 305 | How did you provide complementary feeding to your children? | 1. Bottle feeding  2. Cup feeding  3. Spoon feeding  99.Others/specifies----- |  |
| 306 | When do you wash your hand mostly?  More than one possible answers | 1. Before preparing food  2. After visiting the toilet  3. Before eating food  4. After eating food  5. Before feeding a child including breast milk |  |
| 307 | What did you use usually to wash your hands mostly? (more than one possible answer) | 1. Soap & water  2. Ash & water  3. Water only  99.Others/ specifies |  |
| 308 | Does your child vaccinated for measles?  (If the child is greater than 9 months) | 1. Yes  2. No |  |
| 309 | Does your child supplemented with Vitamin A within the last six months?  (If the child is greater than 6 months) | 1. Yes  2. No |  |

**Observation check list on some environmental and behavioral factors to be completed by data collectors at the end of interview**

| **Code** | **Questions** | **Yes** | **No** |
| --- | --- | --- | --- |
| 1 | Is the latrine functional? |  |  |
| 2 | Is the latrine has hand washing facility? |  |  |
| 3 | Are there feces around the pit hole of the latrine? |  |  |
| 4 | Are there any feces in the compound? |  |  |
| 5 | Are you keeping domestic animals with you in the same house? |  |  |

**Sources:** Muluken D, Abera K, Worku T.Predictors of under-five childhood diarrhea : Mecha District, West Gojam, Ethiopia. Ethiop. J. Health Dev. 2011;25(3):192-200, Bezatu M, Yemane B, Alemayehu W. Prevalence of diarrhea and associated risk factors among children under-five years of age in Eastern Ethiopia: A cross-sectional study. Journal of preventive medicine 2013; 3(7):446-453, and Dessalegn M, Kumie A, Worku W. Predictors of under-five childhood diarrhea: Mecha District, West Gojam, Ethiopia. Ethiop.J. Health Dev 2011; 25(3):192-200.
